# Psychological and Behavioral Predictors of Vaccine Efficacy: Considerations for COVID-19

**DOI:** 10.1177/1745691621989243

**Published:** 2021-03

**Authors:** Annelise A. Madison, M. Rosie Shrout, Megan E. Renna, Janice K. Kiecolt-Glaser

**Affiliations:** 1 The Institute for Behavioral Medicine Research, The Ohio State University College of Medicine; 2 Department of Psychology, The Ohio State University; 3 The Comprehensive Cancer Center, The Ohio State University College of Medicine; 4 Department of Psychiatry and Behavioral Health, The Ohio State University College of Medicine

**Keywords:** depression, stress, vaccine efficacy, COVID-19, SARS-CoV-2, health behaviors

## Abstract

Severe acute respiratory syndrome coronavirus 2 (SARS-CoV-2) vaccine candidates are being evaluated, with the goal of conferring immunity on the highest percentage of people who receive the vaccine as possible. It is noteworthy that vaccine efficacy depends not only on the vaccine but also on characteristics of the vaccinated. Over the past 30 years, a series of studies has documented the impact of psychological factors on the immune system’s vaccine response. Robust evidence has demonstrated that stress, depression, loneliness, and poor health behaviors can impair the immune system’s response to vaccines, and this effect may be greatest in vulnerable groups such as the elderly. Psychological factors are also implicated in the prevalence and severity of vaccine-related side effects. These findings have generalized across many vaccine types and therefore may be relevant to the SARS-CoV-2 vaccine. In this review, we discuss these psychological and behavioral risk factors for poor vaccine responses, their relevance to the COVID-19 pandemic, as well as targeted psychological and behavioral interventions to boost vaccine efficacy and reduce side effects. Recent data suggest these psychological and behavioral risk factors are highly prevalent during the COVID-19 pandemic, but intervention research suggests that psychological and behavioral interventions can increase vaccine efficacy.

Even the best vaccines do not work for everyone. Some vaccines, such as the measles vaccine, are highly efficacious, reducing infection rates by about 98%. The U.S. Food and Drug Administration (2020) said that it will approve any severe acute respiratory syndrome coronavirus 2 (SARS-CoV-2) vaccine that reduces coronavirus-19 disease (COVID-19) cases by at least 50% compared with the placebo (i.e., 50% efficacy), a relatively low bar. Initial reports suggest that the Pfizer–BioNTech and Moderna vaccines are ~95% efficacious (e.g., Mahase, 2020), which is highly impressive given the condensed timeline for vaccine development. However, the AstraZeneca vaccine is less efficacious, with only 62% efficacy among those given two full vaccine doses and 90% in the subset given a half dose followed by a full one (Knoll & Wonodi, 2021). Effectiveness, or how much the vaccine reduces infection rates in the “real world”—outside of the highly controlled clinical-trial setting—is often lower than the initial efficacy rate. Moreover, these reported efficacy rates are based on a relatively short follow-up period, and it is unknown how the vaccine will perform over time. Although vaccine efficacy depends heavily on vaccine-related factors, characteristics of the vaccinated also matter. This review focuses on the latter, given that some of these characteristics, such as stress, are rampant during the pandemic and can not only reduce vaccine efficacy but also promote more immediate and transient side effects, such as fatigue and low mood; however, many of these factors are modifiable and thus may be important intervention targets as the world prepares for widespread immunization.

Psychological, social, and behavioral factors can substantially affect the immune system’s vaccine response. Our lab spearheaded this line of research in the early 1990s with the initial observation that psychological factors shaped the antibody responses to vaccines, even in young and healthy people (Glaser et al., 1992). Since then, a plethora of studies have helped to clarify the psychological factors and poor health behaviors that increase the risk of nonresponsiveness to vaccines. We will review these findings and discuss their relevance to the COVID-19 pandemic, following a brief summary of the immune system’s multifaceted response to vaccines.

## The Immune System’s Immediate and Delayed Vaccine Response

All vaccines challenge the immune system. Inflammatory markers rise within hours of vaccination—thanks to the immediate and nonspecific *innate immune response*, which can produce side effects such as lethargy, malaise, and irritability. As the first prong of the immune response, the inflammatory response usually lasts a few days but can be prolonged in some individuals, such as those who are depressed (Glaser et al., 2003). The *adaptive immune system* mounts the second prong of the immune response. It targets unique vaccine components and therefore takes longer to launch. Vaccines are designed to give the adaptive immune system a lasting memory of viral or bacterial components so that it can quickly and effectively respond when confronted with the actual pathogens. The adaptive immune system responds to the vaccine through (a) T cell multiplication, which can be programmed to identify and kill cells that contain the pathogen (i.e., the cell-mediated response), and (b) B cell production of antibodies, or proteins that neutralize viruses and bacteria.

One critical factor that modulates this response is whether the vaccine recipient has previously encountered the antigen—the protein on the surface of pathogen—either via infection or vaccination. If so, the body mounts a faster and fiercer antibody response—the *secondary immune response*—than it did during the first encounter (i.e., primary immune response). One limitation of this literature is that some studies do not fully account for prior exposure, making it difficult to decipher whether the primary or secondary immune response is reported (Cohen et al., 2001). However, failure to account for prior exposure can mask the magnitude of the impact of stress. Especially among older adults, it is often safe to assume that they have already encountered certain antigens and therefore mount a secondary immune response (Cohen et al., 2001). This is a key consideration for the SARS-CoV-2 vaccine, given that around 10% of Americans had prior exposure as of September 2020 (Bajema et al., 2020). Many more have had exposure to other coronaviruses, which may influence immune responses to SARS-CoV-2 (Poland et al., 2020), and some of the current vaccine candidates require multiple doses.

The studies we will review typically report the antibody response, rather than cellular response, but as stated above, antibody release is just one facet of the adaptive immune system’s response. Because the SARS-CoV-2 virus is novel, it is not yet known how different immune cells and antibodies protect against infection. Cell-mediated (T cell) immunity may play an important role in preventing COVID-19 reinfection because antibody levels naturally wane months after infection (Dan et al., 2020). Even so, there is little evidence that people have robust cell-mediated immunity in the absence of an antibody response, and in one preprint still undergoing peer review (Zuo et al., 2020), the peak antibody response aligned with the T cell response. Thus, antibody titers (levels) may be an important early indicator of lasting immunity (Zuo et al., 2020). Indeed, the antibody titer is considered to be a clinically significant biomarker of protection against SARS-CoV-2 (Poland et al., 2020), although it is not the only such biomarker. It is well established that COVID-19 patients have a highly heterogenous antibody response with greater disease severity associated with more antibody, which itself predicts clinical outcomes (W. Tan et al., 2020). Antibody levels remain highly disparate months later: One study reported a 200-fold difference in SARS-CoV-2 antibody levels 6 months after infection (Zuo et al., 2020)—important variability that may map on to patient characteristics.

## Stress, Depression, and Other Psychological Factors

Before our lab’s vaccine studies, there was already evidence that stress affected various aspects of immune function, but the clinical relevance was unclear. Vaccination is not only beneficial for participants and directly applicable to clinical settings but also a helpful paradigm to assess the immune system’s ability to respond to pathogens, given that everyone receives the same standardized dose but responses can vary widely. In our first study, we administered the standard series of three hepatitis B inoculations over 6 months to medical students, each on the third of 3 days of stressful academic examination blocks (Glaser et al., 1992). After the first dose, 25% of the students developed an antibody response to hepatitis B. These early responders had lower perceived stress and anxiety than those who responded later. Intriguingly, earlier and later responders’ stress, anxiety, and social support had not differed across the earlier academic year, suggesting that their divergent vaccine responses were related to the more proximal stressful examination period. One year later, these findings were replicated (Jabaaij et al., 1993). Subsequent research among healthy young adults revealed that self-rated stress levels in the 10 days after vaccination may be more influential for the antibody response than stress in the prior 2 days, and stress-related sleep loss may be a primary culprit (Miller et al., 2004).

We expanded on this work by looking at vaccine responses in older adults because stress-related immune dysregulation is most pronounced among the elderly (Glaser & Kiecolt-Glaser, 2005). The weakened aging immune system is preoccupied with keeping at bay a whole host of pathogens accumulated throughout the lifetime (Franceschi et al., 2000), so it is less responsive to new immune challenges such as vaccines. We first compared the influenza virus vaccine responses of caregivers of spouses with long-term dementia and age-, sex-, and socioeconomically matched noncaregivers (Kiecolt-Glaser et al., 1996). Caregiving, especially for someone with dementia who requires more intensive and round-the-clock care, is a chronic stressor, often leading to a reduced social network, disengagement from hobbies, and increased risk for anxiety and depressive disorders. Within 4 weeks, only 38% of caregivers had a clinically significant antibody response to the vaccine—defined as a four-fold increase in antibody titers (levels) within 4 weeks—whereas 66% of noncaregivers responded; older age accentuated these differences. In another study, caregivers’ higher cortisol levels helped to explain this disparity (Vedhara et al., 1999). Our caregivers also had poorer cell-mediated vaccine responses, which may be more consequential for older adults’ immune functioning than antibody responses (Lang et al., 2010). Our findings from 1996 are particularly noteworthy because we included only participants who had received an influenza virus vaccine the previous year, which can influence the antibody response to the current vaccine because certain components of the vaccine are often repeated from one year to the next, and there is cross-reactivity among some influenza strains. In addition, we found that caregiving status was associated with both major facets of the adaptive immune response: antibodies and T cells. Subsequent studies in our lab revealed that caregivers’ weak immune response to the influenza virus vaccine persisted long after their spouses’ death (Glaser et al., 1998).

We then investigated whether these findings among caregivers would generalize to a pneumococcal pneumonia vaccine, which protects against a bacterial infection rather than a virus. Note that the immune system’s response to this type of vaccine is independent of T cells and therefore no memory B cells are produced, which means that the body launches a primary immune response even upon repeated exposure. Current and former caregivers and control subjects did not have different antibody responses 2 weeks or 1 month after vaccination, but current caregivers were unable to maintain their antibody levels, with a relative decline 3 and 6 months after vaccination (Glaser et al., 2000). Thus, caregiving stress ultimately eroded the initial vaccine antibody response, perhaps rendering caregivers more susceptible to infection. Similar findings among younger adults also indicate that psychological factors such as stress and social support shape immune responses to bacterial vaccines (Gallagher et al., 2008, 2009b).

Even younger caregivers have poorer antibody responses to vaccines. Parental caregivers of children with developmental disabilities had lower antibody responses to the influenza virus vaccine, adjusting for baseline antibody levels, (Gallagher et al., 2009a) and pneumococcal pneumonia vaccine (Gallagher et al., 2009b), which evokes a primary immune response, compared with parents of typically developing children. These findings indicate that intensive caregiving—even among young people—can increase susceptibility to both bacterial infections (Gallagher et al., 2009b; Glaser et al., 2000) and viral infections (Gallagher et al., 2009a; Glaser et al., 1998; Kiecolt-Glaser et al., 1996).

Among caregivers, psychological distress and negative thought patterns may influence vaccine responsiveness. Spouses and children of community-dwelling patients with Alzheimer’s disease who had greater perceived stress and depressive symptoms had smaller antibody increases following a tetanus vaccine, even after adjusting for baseline antibody levels, which were already providing clinical protection for most of these caregivers (Li et al., 2007). Likewise, among caregivers, negative repetitive thought predicted greater postvaccination depression and lower antibody titers, again controlling for baseline titers (Segerstrom et al., 2008).

In sum, across different vaccine types, both long-term stressors (e.g., caregiving) and short-term stressors (e.g., an academic examination) can impair vaccine responses—particularly the antibody response, which is a primary endpoint in many studies, but there is also some evidence that the cell-mediated response is weaker (Glaser et al., 1992; Kiecolt-Glaser et al., 1996). However, it is notable that very brief (e.g., 10 min) stressors with a clear endpoint that occur immediately after vaccination can ultimately enhance antibody responses (e.g., Brydon, Walker, Wawrzyniak, Chart, & Steptoe, 2009) but may trigger side effects, as discussed below. Distressed individuals’ poorer vaccine responses can persist long after the vaccine administration (e.g., Glaser et al., 2000) and long after the end of the stressor (e.g., Glaser et al., 1998). In particular, our finding that caregivers’ primary antibody responses were not initially lower 2 weeks and 1 month after a pneumococcal pneumonia vaccine but then declined (relative to noncaregivers) 3 and 6 months after vaccination (Glaser et al., 2000) may be particularly relevant to the SARS-CoV-2 vaccine because it (a) showed that stress can affect the primary immune response and (b) suggests that stress may erode antibody levels over time. For a significant subset of the population, the SARS-CoV-2 vaccine will be the first time they encounter the antigen, in which case the primary immune response is relevant. In addition, it is not known how long the SARS-CoV-2 vaccine candidates will protect recipients from infection. Although the current leading vaccine candidates have generally achieved high efficacy, it is possible that vaccine recipients’ chronic stress may lessen this response over time, thus necessitating more frequent vaccination to maintain immunity.

Another line of evidence shows that both state and trait psychological factors may affect the shorter term innate immune response to vaccination, thereby helping to determine the number and severity of postvaccination side effects people experience. When it comes to trait psychological factors and chronic stress, the research is primarily correlational (e.g., Glaser et al., 2003). However, there is compelling experimental evidence about acute stress from a double-blind, randomized, placebo-controlled trial in which participants were assigned to one of four conditions: typhoid vaccine/rest, placebo vaccine/rest, typhoid vaccine/stress, placebo vaccine/stress (Brydon, Walker, Wawrzyniak, Whitehead, et al., 2009). Like the pneumococcal pneumonia vaccine, the typhoid vaccine also triggers a primary immune response regardless of prior exposure. Upon vaccination, participants either rested or completed 10 min of mentally challenging tasks—a Stroop task and a speech task. Even this brief stressful period amplified the inflammatory response to the vaccine, and participants had a larger increase in negative mood after the stressor if they had received the typhoid vaccine rather than the placebo (Brydon, Walker, Wawrzyniak, Whitehead, et al., 2009), demonstrating that stress and vaccination can have synergistic effects. The same lab later reported that among those with high levels of trait optimism, these effects were buffered (Brydon, Walker, Wawrzyniak, Chart, & Steptoe, 2009). Thus, the interplay between state and trait psychological factors may contribute to postvaccination side effects.

Depression alters multiple facets of the vaccine responses. Many depressed individuals’ immune systems are dysregulated even before receiving a vaccine, as evidenced by heightened levels of inflammation (Kiecolt-Glaser et al., 2015). This chronic inflammation may interfere with the vaccine response (Vukmanovic-Stejic et al., 2018). Indeed, unmedicated depressed patients—all with prior exposure to varicella zoster—had lower cell-mediated responses to a varicella zoster virus vaccine than both depressed individuals taking antidepressants and nondepressed individuals, suggesting that they may be at an increased risk for a herpes zoster recurrence (i.e., shingles; Irwin et al., 2013). Among patients undergoing hemodialysis, those with more depressive symptoms had a lower antibody response to the hepatitis B vaccine (Afsar et al., 2009). Adding fuel to the fire, depressive symptoms may amplify and prolong the acute inflammatory response to a vaccine (Glaser et al., 2003). Chronically elevated inflammation reduces the body’s ability to fight infections, and it also accelerates the aging of the immune system, a process called “inflamm-aging” (Franceschi et al., 2000).

Other psychological factors that predicted lower antibody responses to vaccination included high trait negative affect (Marsland et al., 2001), low trait positive affect (Marsland et al., 2006), high neuroticism (Morag et al., 1999; Phillips et al., 2005), and low self-esteem (Morag et al., 1999). Not surprisingly, these dispositional factors increase distress as well as risk for depression.

### Psychological health during the COVID-19 pandemic

These studies from our lab and others have demonstrated how depression and psychological stress—even just a few days before or after the vaccine—can be a powerful and robust predictor of the immune system’s innate and adaptive response. Unfortunately, distress is integral to the COVID-19 pandemic; in fact, in one U.S. sample, the fear of COVID-19 itself, termed “coronaphobia,” drove depression and generalized anxiety, even after adjusting for sociodemographic factors and other psychological vulnerability factors such as neuroticism (S. A. Lee et al., 2020). In another large representative U.S. sample, those with elevated COVID-19 fearfulness were at a particularly high risk for clinically significant depressive symptoms (Fitzpatrick et al., 2020). Ironically, fear of COVID-19 itself may lessen a vaccine’s ability to confer immunity against the virus.

The prevalence of psychiatric symptoms and clinical diagnoses have increased during the worldwide pandemic. According to the U.S. Census Bureau, adults in April and May 2020 had triple the likelihood of screening positive for either a depressive disorder, anxiety disorder, or both compared with adults surveyed in early 2019; in fact, during the pandemic, one in three U.S. adults screened positive for one or both disorder types (Twenge & Joiner, 2020). Between April and May 2020, the prevalence of anxiety declined whereas depression rose (Twenge & Joiner, 2020). In a large representative U.S. sample, the average depressive symptom score was almost one point above the cut score used to identify clinically significant depressive symptoms, reflecting widespread distress (Fitzpatrick et al., 2020). In addition, those who reported food insecurity were particularly at risk (Fitzpatrick et al., 2020), which is concerning given that low socioeconomic status is related to COVID-19 severity (Raifman & Raifman, 2020). Likewise, during the COVID-19 outbreak in China in February 2020, an online survey found that prevalence rates for clinically significant generalized anxiety symptoms, depressive symptoms, and poor sleep quality among the general population were 35%, 20%, and 18%, respectively (Huang & Zhao, 2020). This research suggests that elevated stress, depression, and anxiety are more prevalent during the COVID-19 pandemic, and certain demographics, such as those who are more fearful of COVID-19 or those with lower socioeconomic status, are particularly likely to experience these symptoms—as well as reduced vaccine efficacy.

The above research also demonstrates that both state and trait psychological factors may help determine the prevalence and severity of vaccine-related side effects. For example, experiencing an acute stressful event immediately after vaccination may worsen side effects (Brydon, Walker, Wawrzyniak, Whitehead, et al., 2009). The possibility of SARS-CoV-2 vaccine-related side effects is one factor determining U.S. adults’ willingness to be vaccinated (Reiter et al., 2020). To whatever extent possible, reducing stress exposure around the time of vaccination may help to reduce the likelihood of bothersome side effects (Brydon, Walker, Wawrzyniak, Whitehead, et al., 2009).

## Loneliness and Social Support

Like stress, loneliness can impair immune function, even altering vaccine responses in young and healthy people. In one study among undergraduate students, social dysfunction predicted lower antibody levels following a meningitis C conjugate vaccine (Burns et al., 2002). Another study found that lonelier undergraduate students in the first semester of their first year had lower antibody levels 1 and 4 months after their first-ever influenza vaccination, and a small social network (i.e., 4–12 people contacted in the past month) magnified the inadequate response (Pressman et al., 2005). Lonelier individuals did not have a poorer antibody response when their social network size was large (19–20 members), suggesting that contact with many people may provide some protection even if it is not subjectively satisfying (Pressman et al., 2005). Moreover, those who were lonely felt more overwhelmed and stressed, which contributed to their weaker antibody responses (Pressman et al., 2005), demonstrating that the elevated stress levels of those without an adequate social buffer has immune consequences. Social support also played a central role in our first vaccine study (Glaser et al., 1992): Although self-reported support was initially unrelated to total immune response (i.e., a T cell and B cell summary score), it explained 13% of the variance in total immune response after the third hepatitis B inoculation—an even better predictor than their immune function a few months earlier (Glaser et al., 1992).

Social networks contract with age as older adults prioritize relationships that are the most meaningful (Carstensen, 1992). Therefore, emotional closeness with significant others increases, and a smaller social network does not necessarily foster loneliness (Carstensen, 1992). In fact, subjective well-being rises in later life (Jivraj et al., 2014). Vaccine responsiveness may reflect this age-related shift in social goals. In an elderly sample, bereavement in the year before vaccination predicted a poorer antibody response, whereas those who were married and had high marital satisfaction had a stronger antibody response (Phillips et al., 2006). These findings demonstrate the differential impact of various interpersonal stressors throughout the life span, suggesting that social network size is especially relevant for younger adults, whereas loss of a spouse may be more immunologically relevant (and common) for older individuals.

### Loneliness and social support during the COVID-19 pandemic

During the pandemic, physical-distancing measures reduced face-to-face contacts. The social networks of a cohort of students in Switzerland assessed during the pandemic were smaller than a prepandemic cohort, which predicted worsening mental-health trajectories (Elmer et al., 2020). Beyond social-network size, relationship depth and quality also matter. Drawing on existing relationship theory and research, a recent conceptual article predicted that COVID-19-related stress would amplify destructive dyadic processes, such as hostility and withdrawal, and that certain preexisting contextual factors (e.g., social status, age, mental health) could buffer or bolster this link (Pietromonaco & Overall, 2020), but further empirical work is needed. Zooming out from romantic relationships, recent data show high and increasing rates of loneliness during the U.S. outbreak (Killgore et al., 2020)—although another U.S. study failed to find large increases in loneliness (Luchetti et al., 2020). Overall, the number of face-to-face contacts has decreased during the pandemic, but it is possible that the quality and depth of specific relationships have improved. Even so, individuals who are lonely or socially isolated remain at risk for insufficient vaccine responses that do not confer immunity.

## Health Behaviors Matter

Stressed individuals often have poor health behaviors, such as smoking, eating a low-quality diet, having poor sleep habits, being sedentary, and overusing alcohol. At more extreme levels, health behaviors may have direct associations with vaccine responses or may synergistically interact with stress to predict vaccine response (e.g., Segerstrom et al., 2012).

### Cigarette smoking

Smoking depresses the antibody response to hepatitis B vaccination, as shown in multiple older studies (e.g., Struve et al., 1992; Winter et al., 1994). In fact, a recent meta-analysis found that, compared with nonsmokers, smokers had 1.53 times the risk of nonresponse to the hepatitis B vaccine (Yang et al., 2016). Chronic inflammation may link smoking with a poorer vaccine response (Younas et al., 2017).

### Nutrition

Dietary components and nutritional status are relatively unexplored in relation to vaccine responses among healthy adults. Much of the research in this domain centers on populations in which undernourishment is common: children in developing countries and older adults. Malnourished children are generally able to mount a sufficiently protective immune response after vaccination, but the extent and duration may be less than ideal (Prendergast, 2015). Across several studies, deficiencies in protein, vitamins A and D, iron, and zinc had little to no effect on vaccine response in children (Savy et al., 2009).

Although a single nutrient or nutrient deficiency may have little impact on vaccine response, overall diet may be an important consideration (Butler & Barrientos, 2020). For instance, the Western diet, high in fat, refined sugars, and processed foods, is responsible for an epidemic of chronic inflammation and obesity (Christ et al., 2019). Inflammation is higher among the obese, in part because fat cells can themselves increase inflammatory signaling, which reduces the immune system’s ability to mount an effective response to subsequent immune challenges (Park et al., 2014). In addition, diet powerfully shapes the gut microbiota (David et al., 2014), and the gut microbiota also determine vaccine responses (Harris et al., 2018; Oh et al., 2014). As a primary example, dietary fiber intake promotes a greater abundance of bacteria (e.g., *Bifidobacteria*) that produce short-chain fatty acids, which can boost antibody responses (Huda et al., 2014; Lynn & Pulendran, 2018).

### Sleep

Sleep substantially affects immune function. People who are regularly sleep deprived are at great risk not only for vaccine nonresponsiveness but also for severe illness. The relationship between disturbed sleep and lower antibody responses has been documented in both cross-sectional studies (Burns et al., 2002) and research with experimentally induced sleep restriction (Spiegel et al., 2002). For the latter, healthy young men who normally spent between 7.5 and 8.5 hr in bed were restricted to 4 hr per night in bed for 6 nights, which then lengthened to 12 hr per night for 7 nights to recover from the deprivation. On the morning after the fourth short night of sleep, they received an influenza virus vaccine. Despite the period of sleep recovery, these individuals had lower antibody production than their normally rested peers 10 days after vaccination, even accounting for baseline antibody titers. The notable between-subjects variation within each group suggested that sleep deprivation does not uniformly impair antibody responses. By three to four weeks after vaccination, their antibody levels no longer differed from those of their peers (Spiegel et al., 2002).

In another sleep-restriction study, young adults who were allowed a normal night’s sleep after a hepatitis A vaccine had double the antibody response 1 month later compared with those who were not allowed to sleep for 36 hr after vaccination (Lange et al., 2003). The enhanced antibody response mirrored the normal sleepers’ increased release of immune-stimulating hormones on the night and day after vaccination and their lower levels of stress hormones. It is noteworthy that none of these participants had been infected with hepatitis A and had very low antibody titers before vaccination, suggesting that sleep deprivation modulates the primary immune response. The same research group later used a similar sleep-restriction paradigm with a hepatitis A vaccination and found marked differences in cell-mediated adaptive immunity—effects that were still evident 1 year after vaccination—and concluded that sleep fosters improved immune memory (Lange et al., 2011).

Among midlife adults, sleep duration also matters for vaccine efficacy. In one study, among participants who had no serological evidence of prior hepatitis B exposure, those who reported less sleep—especially on the two nights before a hepatitis B vaccination—had lower antibody titers 1 and 4 months later (Prather et al., 2012). Likewise, sleep duration, measured objectively via actigraphy and averaged the three nights before and three nights after the first hepatitis B inoculation, predicted subsequent antibody responses and clinical protection status after the second and third shots (Prather et al., 2012). In fact, each additional hour of sleep tracked with a 56% increase in antibody levels. Taken together, this evidence suggests that shorter sleep duration lowers antibody responses—at least initially (e.g., Spiegel et al., 2002)—and fosters longer lasting deficiencies in cell-mediated immunity across a variety of vaccines and regardless of prior exposure (Lange et al., 2003, 2011; Prather et al., 2012; Spiegel et al., 2002).

### Sedentariness and physical activity

Physical activity promotes a strong immune system and better vaccine responses. Physically fit elderly people had better antibody immune responses, but not cell-mediated immune responses, to tetanus and influenza virus vaccines compared with their less-fit peers (Keylock et al., 2007). Accelerometer data collected from elderly Singaporean Chinese women showed that those who walked more (> 18,509 steps/day) for 2 weeks after an influenza virus vaccination had greater innate immune activation 2 days after vaccination, larger adaptive immune responses 1 week after vaccination, and greater antibody responses after a second vaccination than their less active peers (< 10,927 steps per day; Choon Lim Wong et al., 2019). Likewise, physically active older men who had done regular aerobic exercise at least three times per week for at least 2 years, had higher antibody responses to a novel immune challenge compared with their peers who had not regularly exercised for at least the past 2 years (Smith et al., 2004). Adults 62 years and older who engaged in at least 20 min of vigorous exercise (i.e., intense enough to cause large increases in heart rate, breathing, and sweating that makes it somewhat difficult to have a conversation) three or more times per week had greater antibody and cellular responses to an influenza virus vaccine compared to moderately active or sedentary older adults (Kohut et al., 2002). These results implicate physical activity in vaccine responses to both novel (Smith et al., 2004) and familiar (Choon Lim Wong et al., 2019; Kohut et al., 2002) antigens.

### Alcohol consumption

Moderate alcohol use may not weaken the immune response to a vaccine. In fact, moderate use may even increase responses: Rhesus monkeys that had moderate alcohol intake for 7 months had enhanced antibody and cell-mediated response to a booster shot, but those with long-term consumption of high levels of alcohol had weaker responses (Messaoudi et al., 2013). Research among humans similarly suggests that moderate alcohol use bolsters immune function: A greater number of alcoholic drinks (up to three or four per day) is associated with reduced risk of developing a cold upon exposure to the virus (Cohen et al., 1993). However, alcohol overuse and abuse clearly harms immune function: Half of those who had high long-term alcohol intake and liver cirrhosis had no detectable antibody titers to hepatitis B after receiving three or four doses (Degos et al., 1986). All of those without cirrhosis had a detectable but relatively low antibody response (Degos et al., 1986). Thus, the relationship between alcohol use and immune response to vaccine may be an upside-down U-shaped curve, with better responses up to a certain level of alcohol use and then diminishing responses past that point.

### Health behaviors during the COVID-19 pandemic

The COVID-19 pandemic is undermining health behaviors. A recent commentary called attention to the well-established link between stress and problematic drinking behavior, suggesting that the pandemic will result in a spike in substance addiction that would further strain treatment and rehabilitation services (Clay & Parker, 2020). Indeed, data suggest that alcohol use is more prevalent during the COVID-19 pandemic. For instance, U.S. alcohol sales increased 54% in late March 2020 compared with the same time the year before, whereas online sales boomed—rising by 500% in late April (Nielsen Company, 2020).

In terms of sleep duration, survey data from the Chinese general public during the February 2020 COVID-19 outbreak found that at least 20% met criteria for clinical insomnia, and the same proportion spent more than 1 hr awake in bed (Lin et al., 2021). Moreover, females, young people, and those at greater risk for SARS-CoV-2 exposure during the outbreak (e.g., first-line hospital workers) had more severe insomnia (Lin et al., 2021). During strict lockdown measures, those with psychiatric disorders before the pandemic were at even greater risk for elevated depressive and anxiety symptoms as well as insomnia (Hao et al., 2020). Insomnia is such a central problem during the pandemic that the European Cognitive-Behavioral Therapy Academy task force released practical recommendations for dealing with sleep problems during at-home confinement (Altena et al., 2020). The task force recommended using social media to share anxieties as well as positive “distractions,” limiting exposure to COVID-19 news, and keeping a regular sleep-wake time, as well as other sleep-hygiene tips.

Overeating is common during stressful times, particularly more energy-dense foods, thus fueling weight gain (Razzoli et al., 2017). After 1 month of enforced lockdown in Northern Italy, obese individuals reported gaining an average of 3.3 pounds, which was associated with unhealthy food consumption, increased boredom, higher anxiety and depressive symptoms, and lower exercise (Pellegrini et al., 2020). There are also fears that pandemic-related school closures will fuel poor eating behavior and weight gain in children (Rundle et al., 2020). In addition, undernourishment, common among older adults, may jeopardize the elderly’s vaccine response.

Concerning physical activity, the World Health Organization recommends 150 min per week of moderate-intensity exercise (e.g., walking) or 75 min per week of vigorous-intensity exercise (e.g., jogging). Achieving these guidelines is associated with a 17% lower risk of cardiovascular events, a 23% lower risk of cardiovascular mortality, and a 26% lower occurrence of type 2 diabetes (Wahid et al., 2016). These outcomes are relevant to COVID-19 patients, who are at risk for cardiovascular morbidity and mortality (Wahid et al., 2016). The American company Fitbit just released data from 30 million users that show 7% to 38% reductions in average step counts across most countries in late March (Fitbit Staff, 2020), but more peer-reviewed empirical evidence is needed.

Taken together, the COVID-19 pandemic and related stress promote poor health behaviors that in turn worsen mental and physical health in a vicious cycle, ultimately driving weight gain. Ironically, the pandemic lifestyle could lower the efficacy of a SARS-CoV-2 vaccine.

## Treatment

Intervention research corroborates the observed links between psychological and behavioral factors and vaccine responses discussed above. Specifically, these findings indicate that psychological and behavioral interventions may boost immune responses to vaccines. Interventions vary in type, dose, and duration, and thus they can be selected on the basis of individual needs.

Psychological interventions as possible vaccine adjuvants include massage, meditation/mindfulness, expressive writing, and stress management (Vedhara et al., 2019). However, results are inconsistent, probably because of sample age differences, vaccine type, intervention type, and varying times between vaccination and intervention. improved antibody responses were observed in four out of seven randomized controlled trials (Vedhara et al., 2019). The median successful intervention length was six sessions and a total of 280 min (Vedhara et al., 2019). In particular, an 8-week cognitive-behavioral stress-management intervention among elderly spousal dementia caregivers enhanced antibody responses to influenza virus vaccination (Vedhara et al., 2003); 50% of caregivers who received the intervention responded to vaccination, whereas only 7% of caregivers and 29% of noncaregiving control subjects not in the intervention responded. The mechanism remains unclear because caregivers maintained higher levels of distress throughout the intervention, compared with control subjects, and there were no between-groups differences in cortisol. Likewise, in a randomized, controlled trial, an 8-week mindfulness intervention among healthy employees in a work environment boosted antibody responses to an influenza vaccine between 4 and 8 weeks after vaccination (Davidson et al., 2003). Expressive writing has mixed effects: short writing bouts about personal experiences of racism lowered antibody slopes by 40% to 50% (Stetler et al., 2006), whereas brief writing sessions about a traumatic event increased antibody levels over time (Petrie et al., 1995). Overall, randomized, controlled trials of psychological interventions to boost vaccine efficacy are few and far between, and further work is needed in this domain. In particular, psychological interventions that reliably reduce anxiety and depressive symptoms, such as a full course of cognitive-behavioral therapy, are worthy of further investigation as vaccine adjuvants. In addition, more work is needed to see whether even brief psychological interventions reduce the prevalence and intensity of vaccine-related side effects.

Behavioral interventions have shown promising results. A systematic review on exercise interventions found fairly robust evidence that both short- and long-term exercise interventions can improve immune responses to vaccination, especially among those who are otherwise at risk for poor responses (e.g., sedentary individuals; Pascoe et al., 2014). Note that a 25-min eccentric-exercise protocol targeting the arm muscle at the site of injection performed 6 hr before an influenza virus vaccine improved antibody responses in women and cell-mediated responses in men compared with those who rested quietly (Edwards et al., 2007). Nevertheless, further investigation suggested that such an acute exercise protocol may affect cell-mediated and antibody vaccine responses only when the control group has a poor response (Campbell et al., 2010) and therefore may not be necessary for vaccines that are highly efficacious or among demographics that have robust responses. A similar brief (15 min) exercise protocol reduced days of swelling, fever, and low appetite, compared with a resting condition, among adolescents who received a human papillomavirus vaccine (V. Lee et al., 2018). Among older adults, a 45-min moderate-intensity resistance exercise session immediately before receiving the influenza vaccine reduced vaccine reactions—including pain, redness, or swelling at injection site or other symptom/illness—compared with a resting condition (Bohn-Goldbaum et al., 2020). In terms of longer exercise protocols, 10 months of cardiovascular training among sedentary, community-dwelling older adults did not affect peak antibody levels 3 and 6 weeks after influenza vaccination, but it did promote clinically significant antibody levels 6 months after vaccination, compared with flexibility and balance training (Woods et al., 2009).

Along with exercise, boosting nutritional status via supplementation may result in a better antibody response to vaccines, especially in older adults, who often struggle to meet recommended daily nutritional guidelines, especially if they live alone. In one randomized, controlled trial, elderly individuals who received a complete liquid nutritional supplement containing vitamins and minerals, including antioxidants, had higher antibody levels 1 month after influenza virus vaccine administration compared with those who received a placebo (Wouters-Wesseling et al., 2002). There is little evidence that micronutrient supplementation at the time of vaccination improves responses (Savy et al., 2009). Even so, a randomized, double-blind, placebo-controlled trial among elderly nursing-home residents revealed that zinc supplementation successfully increased serum zinc levels and led to increased T cell multiplication (Barnett et al., 2016), which could bode well for vaccine responsiveness. In addition, preclinical evidence suggests that vitamin A supplementation may have a similar effect (X. Tan et al., 2011). Taken together, there is promising evidence that even short-duration psychological and behavioral interventions can modify the immune system’s vaccine response as well as vaccine-related side effects, but further research is needed to assess (a) the optimal dose and timing of the intervention and (b) whether change in the targeted psychological construct or behavior is the mechanism driving the improved vaccine response.

## Other Virus-Related Considerations

Psychological and behavioral factors interact with the current pandemic in many ways beyond vaccine response. These factors can influence susceptibility to infection on SARS-CoV-2 exposure and willingness to be vaccinated (i.e., vaccine uptake). The psychological vulnerabilities for infection after virus exposure are elegantly reviewed elsewhere (Cohen, 2021) and largely overlap with vulnerabilities for poor vaccine response. Vaccine uptake is also a concern: Recent data indicate that around half of Americans may refuse to receive a SARS-CoV-2 vaccine (Cornwall, 2020). Although vaccine uptake is beyond the scope of this review, psychological factors clearly play a role at this stage as well.

## Conclusions

Work from our lab and many others has identified psychological and behavioral factors as key determinants of the immune system’s response to many different types of vaccines, helping to determine the side-effect profile as well as efficacy. Unfortunately, the COVID-19 pandemic itself has aggravated many of the risk factors for poor vaccine responses, such as stress and sedentariness—although there is mixed evidence on loneliness: One study found stable levels, perhaps indicating some resilience. Overall, these risk factors are so prevalent that if they are not addressed, they could significantly reduce the overall effectiveness of SARS-CoV-2 vaccine candidates. Prior research suggests that psychological and behavioral interventions can improve vaccine responsiveness. Even short-term interventions can be effective. Therefore, now is the time to identify those at risk for a poor immune response and to intervene on these risk factors.

## Transparency

*Action Editor:* Aina Puce

*Editor:* Laura A. King
